# Mandibular Radiomorphometric Indices and Tooth Loss as Predictors for the Risk of Osteoporosis using Panoramic Radiographs

**DOI:** 10.3290/j.ohpd.a45081

**Published:** 2020-09-04

**Authors:** Ray Tanaka, Tatsurou Tanaka, Andy Wai Kan Yeung, Akira Taguchi, Akitoshi Katsumata, Michael M. Bornstein

**Affiliations:** a Senior Clinical Practitioner, Oral and Maxillofacial Radiology, Applied Oral Sciences and Community Dental Care, Faculty of Dentistry, The University of Hong Kong. Contributed to study design, evaluating the images, analysing the data, drafting and writing the manuscript.; b Associate Professor, Division of Oral and Maxillofacial Radiology, Kyushu Dental University, Kitakyushu, Fukuoka, Japan. Contributed substantially to collecting and evaluating the panoramic radiographs and analysing the data.; c Tutor in Radiography, Oral and Maxillofacial Radiology, Applied Oral Sciences and Community Dental Care, Faculty of Dentistry, The University of Hong Kong, Hong Kong. Contributed substantially to developing the study design and revising the manuscript.; d Chair and Professor, Department of Oral and Maxillofacial Radiology, School of Dentistry, Matsumoto Dental University, Shiojiri, Nagano, Japan. Contributed to the study design and statistical analysis, provided expertise concerning osteoporosis and mandibular indices.; e Chair and Professor, Oral Radiology, Asahi University, Mizuho, Gifu, Japan. Provided expertise concerning the automated bone morphometry system.; f Honorary Professor and Clinic Manager, Oral and Maxillofacial Radiology, Applied Oral Sciences and Community Dental Care, Faculty of Dentistry, The University of Hong Kong, Hong Kong; Professor, Department of Oral Health & Medicine, University Center for Dental Medicine Basel UZB, University of Basel, Basel, Switzerland. Contributed to the study design, manuscript preparation and submission.

**Keywords:** mandible, osteoporosis, panoramic radiography, radiomorphometric indices, tooth loss

## Abstract

**Purpose:**

To assess the mandibular cortical width (MCW) and morphology of the mandibular inferior cortex (MIC) on panoramic views from a large sample of males and females in various age groups by using an automated morphometric grading system for assisting osteoporosis screening. Furthermore, possible predictors and concrete cut-off values to identify the risk for osteoporosis were evaluated.

**Materials and Methods:**

MCW, MIC, tooth loss (TL), and alveolar bone loss (ABL) were retrospectively evaluated in 700 panoramic images from dental patients in Hong Kong using commercially available software. To estimate possible predictors for identifying the risk of osteoporosis, age, TL, and ABL were evaluated with the receiver operating characteristic (ROC) curves for each gender separately.

**Results:**

The age groups 60s (sixties), 70s and 80s showed statistically significant gender differences. For example, a smaller MCW and more MIC Class 3 were found in females. Furthermore, females exhibited a statistically significant increase in TL in the age groups 50 years and above. In males, age, TL or ABL did not correlate with MCW, whereas in females it statistically significantly did. Meanwhile, the correlation between ABL and MCW and MIC was weak for both genders. Concrete cut-off values to identify patients at risk of osteoporosis were 60.15 years and 3.5 missing teeth in females, and 72.55 years in males.

**Conclusion:**

Age and tooth loss were related to MCW and MIC in the population investigated. An age of ≥60 as well as more than 3.5 teeth lost seem to be indicators for a risk of osteoporosis in Chinese females based on panoramic views using artificial-intelligence-based software.

Osteoporosis is defined as a skeletal disorder characterised by compromised bone strength due to the loss of bone density and degeneration of bone quality, leading to an increased risk of fracture.^23^ This skeletal disorder causes more than 8.9 million fractures annually, resulting in one fracture every 3 s worldwide.^11^ Early diagnosis and treatment are important to prevent fractures. Bone mineral density (BMD) can be measured as the major parameter for bone strength.^12,37^ However, since osteoporosis progresses without symptoms, a bone fracture is often the first clinical sign of the disease.^23^

The thickness and morphological analysis of mandibular bone in panoramic radiographs may be a useful tool for screening and identifying patients with osteoporosis.^29^ Particularly, usefulness and reliability of the width (MCW) and morphology (MIC) of the mandibular inferior cortex at the mental foramen region have been evaluated and validated in numerous studies.^4,28,30,33,34^

A possible relationship between periodontal disease and osteoporosis has been discussed in several studies.^12,16,20,25,35^ Most of these studies were chiefly concerned females, in particular postmenopausal women, and evaluated the association between skeletal BMD and parameters for the assessment of periodontal health, such as gingival bleeding, periodontal pocket depth, the level of alveolar bone, or loss of attachment and teeth.^12,16,20,25,35^ Meanwhile, only a few studies using MCW and MIC have discussed a potential association between osteoporosis and periodontal disease in males, perimenopausal women, and/or younger individuals.^5,9,27,32^

As MCW and MIC have demonstrated usefulness in screening for osteoporosis using panoramic radiographs, this study hypothesised that it would be possible to identify skeletal osteoporosis through a possible correlation between MCW and/or MIC and the individual periodontal condition. The purpose of this study was to measure and evaluate MCW and MIC in panoramic images from a large sample of males and females in various age groups by using an automated morphometric grading system to assist osteoporosis screening, and to determine whether the subject’s age, gender, tooth loss or alveolar bone loss were associated with these parameters as indicators for osteoporosis. Furthermore, concrete cut-off values identifying the risk of osteoporosis were sought among age, tooth loss and alveolar bone loss parameters.

## Materials and Methods

### Ethical Approval

All procedures performed were in accordance with the ethical standards of the institutional and/or national research committee and with the 1964 Helsinki declaration and its later amendments or comparable ethical standards. The study protocol was submitted to and approved by the local institutional review board (IRB) of the University of Hong Kong / Hospital Authority Hong Kong West Cluster (date of approval; November 9, 2018, approval number; UW 18–567).

### Population Investigated

This retrospective study included 700 panoramic images from patients who had visited the Prince Philip Dental Hospital in Hong Kong from 2015 to 2017 (3-year period). The panoramic images were randomly collected according to the patient dental hospital ID number by using stratified systematic sampling methods. Seven age groups were first established for both males and females, then the panoramic images were grouped according to patient’s age at date of examination. The following images were excluded: from patients with indistinct mental foramina, after orthognathic surgery, genetic/developmental disease affecting teeth and jaws, trauma in the mandible, destructive bone lesion(s), and images with technical errors regarding patient positioning and head alignment. Thus, panoramic images were obtained for exactly 50 subjects in each age group (7 age groups) and for both genders. Therefore, the final number of panoramic views comprised 700 images. All panoramic images were saved in DICOM format for analysis using a customised bone morphometry software programme (MEDIA; Tokyo, Japan)^21,22^ on a monitor with a resolution of 1920 x 1080 pixels (NEC LAVIE, NEC; Tokyo, Japan).

The age groups selected for the present analysis were as follows:

- 20s (twenties): 20–29 years
- 30s: 30–39 years
- 40s: 40–49 years
- 50s: 50–59 years
- 60s: 60–69 years
- 70s: 70–79 years
- 80s: ≥80 years

### Panoramic Image Analysis

#### Evaluation of mandibular radiomorphometric indices

**Fig 1:**
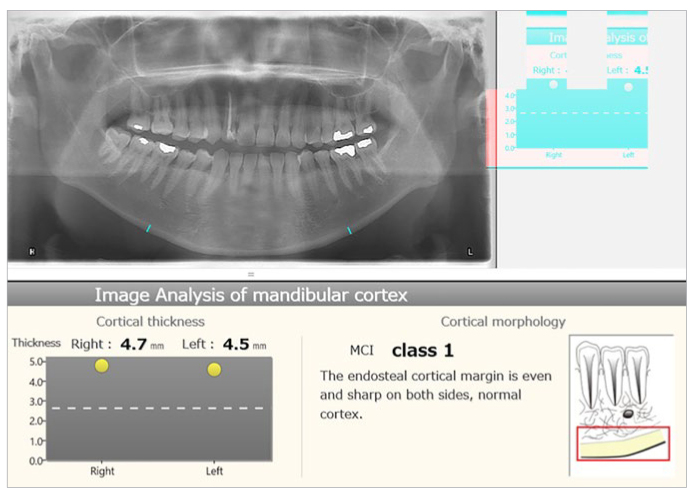
Display of the customised bone morphometry software used (PanoSCOPE, MEDIA) showing results of an image analysis of the mandibular cortex. The MCW is indicated for both sides with blue lines in the panoramic image, and also displayed in mm, and the MCI (MIC class in the present study) is indicated as one value. MCW is indicated by blue lines in the panoramic image, and also displayed in mm.

MCW measurements and the MIC classification were performed using the customised software (PanoSCOPE, MEDIA).^21,22^ The software was developed using role-based training models in artificial intelligence technology. The algorithm used was developed on the basis of hundreds of panoramic radiographs including cases with and without osteoporosis. The system automatically determines mental foramina for analysis of the radiomorphometric indices, and processes the images to output the results of the bilateral measurements for MCW and the MIC classifications. Regarding MIC classification, the software always displayed only the larger code from the results of left and right (Fig 1).

#### Measurement of mandibular cortical width (MCW)

**Fig 2:**
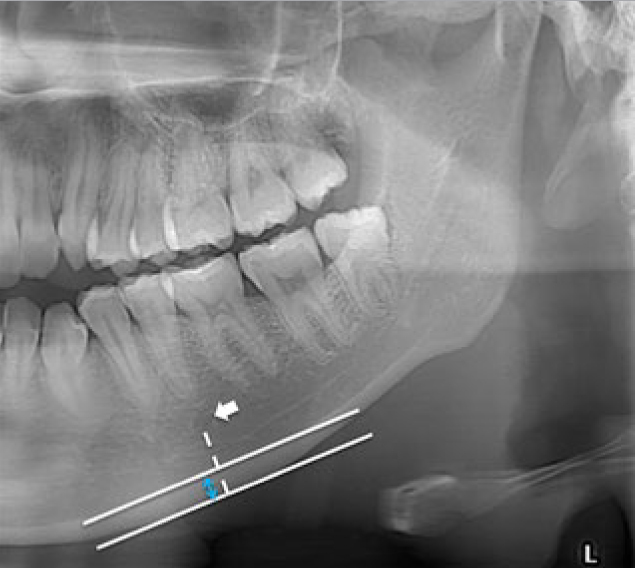
Methodology for the measurement of the MCW at the inferior border of the mandible at the mental region. A line was drawn parallel to the long axis of the mandible and tangential to the inferior border of the mandible. The MCW is the width of the inferior cortex (highlighted in blue) in mm at the site where the line (dotted line) from the mental foramen crosses the two lines perpendicularly.

The cortical width of the lower border of the mandible in the mental foramen region was measured. The method used by the software is shown in Fig 2. MCWs were measured bilaterally, and the mean value was calculated.

#### Classification of the morphology of the mandibular inferior cortex (MIC)

**Fig 3:**
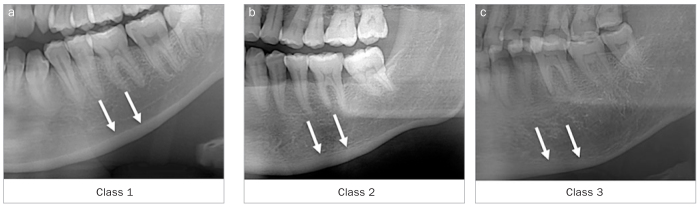
Klemetti’s classification for the morphological analysis of the mandibular inferior cortex (MIC): Class 1 = normal cortex, the endosteal margin of the cortex is even and sharp on both sides (A); Class 2 = the endosteal margin shows semilunar defects (lacunar resorption) with the formation of cortical residues one to three layers thick (B); Class 3 = The cortex is obviously porous with dense endosteal residues (C). White arrows indicate the endosteum site of the mandibular inferior cortex.

The mandibular cortical shape at the mental foramen region was classified into one of three groups by the software according to the method of Klemetti et al^17^ (Fig 3):

- Class 1: normal cortex, the endosteal margin of the cortex is even and sharp on both sides
- Class 2: the endosteal margin shows semilunar defects (lacunar resorption) with the formation of cortical residues one to three layers thick
- Class 3: the cortex is obviously porous with dense endosteal residues.

#### Parameters for periodontal condition and health

All observations for tooth and alveolar bone loss were performed by two certified oral and maxillofacial radiologists (R.T. and T.T.) twice with a time gap of at least 2 weeks to test for intra-observer (repeatability) and inter-observer agreement (reproducibility). In cases of disagreement, the observers discussed the observations to reach a consensus. The findings (tooth loss and alveolar bone loss) were then utilised for further analysis.

- Tooth loss (TL): the total number of missing teeth in the maxilla and mandible, excluding the third molars, was counted in each subject. Root remnants without supporting alveolar bone were counted as ‘tooth loss’. Multi-rooted teeth were regarded as missing when all the roots were missing.
- Degree of alveolar bone loss (ABL): ABL was evaluated at the remaining teeth in the lateral mandibular region including the 1st and 2nd premolars and molars but not wisdom teeth. The side for evaluation, left or right, was determined randomly by the case number in the anonymised data of this study. ABL was classified into the following categories according to the percentages of horizontal and/or vertical bone resorption relative to the root length as measured using a Schei ruler:^26^ 0: no bone resorption; 1: up to 15%; 2: more than 15%, up to 30%; 3: more than 30%.1 The largest code value among all the remaining teeth in the site was assigned whenever applicable. NA was applied to edentulous sites.

### Statistical Analysis

All data were first analysed descriptively. Every analysis was done on the subject level. For further evaluation, differences between gender and age groups regarding TL, MCW, ABL, and MIC were assessed. Non-parametric tests were used to evaluate gender differences, and ANOVA with Tukey’s B post-hoc adjustment were employed for age differences of continuous independent variables (TL and MCW). Categorical independent variables (ABL and MIC) were evaluated with Pearson’s chi-squared test to assess the distribution by gender and age group, and also for evaluating gender and age differences. Pearson’s product-moment correlation coefficient and Spearman’s rank correlation coefficient were calculated for assessing the relationships between age, TL, ABL, MCW and MIC in each gender.

In order to estimate possible predictors for identifying the risks of osteoporosis, age, TL, and ABL were evaluated with receiver operating characteristic (ROC) curves and area under the curve (AUC) for each gender. The diagnostic threshold for risks of osteoporosis in the ROC analysis was set at MIC 3 (Class 3 of MIC classification),^4,28,34^ and a 3-mm MCW (mandibular cortical width of 3 mm).^6^ As a guide for interpretation, the following criteria were applied: non-informative (AUC=0.5), less accurate (0.5<AUC<0.7), moderately accurate (0.7<AUC<0.9), highly accurate (0.9<AUC<1) and perfect tests (AUC=1).^8^ To classify as a possible predictor for identifying risk of osteoporosis, the respective AUC had to be at least moderately accurate. Concrete cut-off values for possible predictors were estimated from ROC curves only for a parameter whose AUC was over 0.7.

For intra- and inter-observer reproducibility of TL and ABL, Cohen kappa values were calculated. All analyses were performed with SPSS (Version 25.0, IBM; Armonk, NY, USA). All p-values were two-sided (two-tailed significance level of 5%).

## Results

### Descriptive Analysis of MCW, TL, MIC and ABL in each Gender and Age Group

A statistically significant gender difference was observed for the average of the MCW values (p < 0.01) in the age groups 60s, 70s, and 80s (Table 1). A statistically significant age difference for MCW was found (p < 0.01) only in females between the age groups 40s and 50s, 50s and 60s, and 70s and 80s. The MCW for females sharply decreased at an age of 50 and above, and differed statistically significantly from males. For the average of TL, gender differences were not observed (Table 1). Meanwhile, a statistically significant age difference was observed for TL in menopausal and postmenopausal females, specifically between the age groups of 50s and 60s, 60s and 70s, and 70s and 80s (p < 0.01). Males showed an age difference only between 60s with the next youngest age group.

**Table 1:** Descriptive data of age, MCW and TL in each gender and age group

Age group	Average age (years)	MCW (mm)			TL (0–28)
		Left	Right	Average of left and right	
Male					
20s	24.7 ± 2.6	4.1 ± 0.8	4.4 ± 1.1	4.3 ± 0.8	0.3 ± 1.1
30s	34.2 ± 3.2	4.3 ± 0.9	4.4 ± 1.0	4.4 ± 0.9	0.4 ± 1.1
40s	45.5 ± 3.0	4.4 ± 1.0	4.6 ± 0.9	4.5 ± 0.9	2.9 ± 4.1
50s	56.0 ± 2.8	4.4 ± 1.0	4.6 ± 1.0	4.5 ± 0.9	4.6 ± 5.5
60s	64.9 ± 3.0	4.6 ± 1.1	4.6 ± 1.2	4.6 ± 1.0 †	7.9 ± 7.1 *
70s	75.6 ± 2.3	4.4 ± 1.1	4.4 ± 1.1	4.4 ± 1.0 †	10.6 ± 7.6
80s	84.3 ± 3.4	4.3 ± 1.4	4.2 ± 1.2	4.2 ± 1.2 †	13.1 ± 8.7
All	55.0 ± 20.3	4.3 ± 1.0	4.5 ± 1.1	4.4 ± 0.9 †	5.7 ± 7.3
Female					
20s	25.1 ± 2.1	4.4 ± 0.9	4.5 ± 0.8	4.5 ± 0.8	0.2 ± 0.7
30s	34.8 ± 3.0	4.5 ± 0.8	4.6 ± 1.0	4.6 ± 0.8	0.8 ± 1.3
40s	45.2 ± 2.7	4.7 ± 1.1	4.7 ± 1.0	4.7 ± 0.9	2.0 ± 2.9
50s	54.9 ± 2.8	4.2 ± 1.1	4.3 ± 1.1	4.2 ± 1.0 *	2.7 ± 2.7
60s	64.6 ± 2.8	3.6 ± 1.3	3.7 ± 1.1	3.6 ± 1.1 †*	6.1 ± 6.2 *
70s	74.8 ± 2.4	3.5 ± 1.4	3.7 ± 1.3	3.6 ± 1.2 †	9.7 ± 7.7 *
80s	84.0 ± 3.8	2.9 ± 1.3	3.2 ± 1.4	3.0 ± 1.2 †*	15.2 ± 8.3 *
All	54.8 ± 20.0	4.0 ± 1.3	4.1 ± 1.2	4.0 ± 1.2 †	5.3 ± 7.2

† Gender difference (p < 0.01) in the same age group. * Age difference (males: p < 0.05; females p < 0.01) from the next youngest age group.

A statistically significant difference in the gender and age distribution of MIC grading was found (chi-squared test, p < 0.01) (Table 2). Statistically significant gender differences for class 1 and class 3 MIC were found in the older age groups. In males, this was more evident in the 80s age group for class 1, and for females, this was more apparent in the 60s, 70s, and 80s age groups for class 3.

**Table 2:** Distribution in MIC classification in each gender and age group

Classification	Class 1		Class 2		Class 3	
Age group	Male	Female	Male	Female	Male	Female
20s	38	39	12	11	0	0
30s	32	31	18	19	0	0
40s	24	26	25	24	1	0
50s	25	25	24	21	1	4
60s	18	10 *	32	31	0 †	9 †
70s	15	12	31	27	4 †	11 †
80s	12 †	1†*	32	32	6 †	17 †
All (%)	164 (46.9)	144 (41.2)	174 (49.7)	165 (47.1)	12 (3.4)	41 (11.7)

Class 1: normal cortex, the endosteal margin of the cortex is even and sharp on both sides. Class 2: the endosteal margin shows semilunar defects (lacunar resorption) with the formation of cortical residues one to three layers thick. Class 3: the cortex is obviously porous with dense endosteal residues. † Gender difference (p < 0.05) in the same age group; *age difference (p < 0.05) from the next youngest age group.

Regarding ABL, no subjects in the groups from 40s to 80s and over in both males and females showed a value of 0 (Table 3). Meanwhile, edentulous cases were not observed in the age groups 20s and 30s in both males and females.

**Table 3:** Distribution in ABL Classification in each gender and age group

Classification	0		1		2		3		NA	
Age group	Male	Female	Male	Female	Male	Female	Male	Female	Male	Female
20s	6 †	14 †	30	27	12	9	2	0	0	0
30s	2	1 *	19	21	20	23	9	5	0	0
40s	0	0	6	11	30	25	13	14	1	0
50s	0	0	3	3	24	27	18	20	5	0
60s	0	0	1	1	15	24	29	24	5	1
70s	0	0	0	1	15	19	19	21	16	9
80s	0	0	1	3	10	16	26 †	11 †	13	20
All (%)	8 (2.3)	15 (4.3)	60 (17.1)	67 (19.1)	126 (36.0)	143 (40.9)	116 (33.2)	95 (27.1)	40 (11.4)	30 (8.6)

0: no bone resorption, 1: up to 15%, 2: more than 15% and up to 30%, 3: more than 30%, NA: edentulous. † Gender difference (p < 0.05) in the same age group; * age difference (p < 0.05) from the next youngest age group.

Intra-observer repeatability and inter-observer reproducibility were high regarding TL (κ = 0.991 and κ =0.988, respectively) and ABL (κ = 0.834 and κ =0.796, respectively).

### Correlation Between Mandibular Radiomorphometric Indices and Age, TL, ABL

The results of Pearson’s product-moment correlation analyses or Spearman’s rank correlation analyses are shown in Table 4. The analyses exhibited that age, TL and ABL were not correlated with MCW in males. Nevertheless, those parameters showed a statistically significant (p < 0.01) correlation with MIC values. For females, a statistically significant (p < 0.01) degree of correlation between MCW and MIC values with age, TL, and ABL was observed.

**Table 4:** Correlation of age, TL, and ABL with mandibular radiomorphometric indices

Parameters		Mandibular radiomorphometric indices	
		MCW	MIC
Age	Male	NS	r = 0.361 #
	Female	r = -0.443 §	r = 0.534 #
TL	Male	NS	r = 0.294 #
	Female	r = -0.347 §	r = 0.437 #
ABL	Male	NS	r = 0.217 #
	Female	r = -0.190 #	r = 0.279 #

§ Pearson product-moment correlation coefficients; # Spearman rank correlation coefficient; all Pearson product-moment correlation coefficient and Spearman rank correlation coefficients are at p = 0.01. NS: not statistically significant.

### Diagnostic Efficacy of Age, TL and ABL to Assess Risk of Osteoporosis

**Fig 4:**
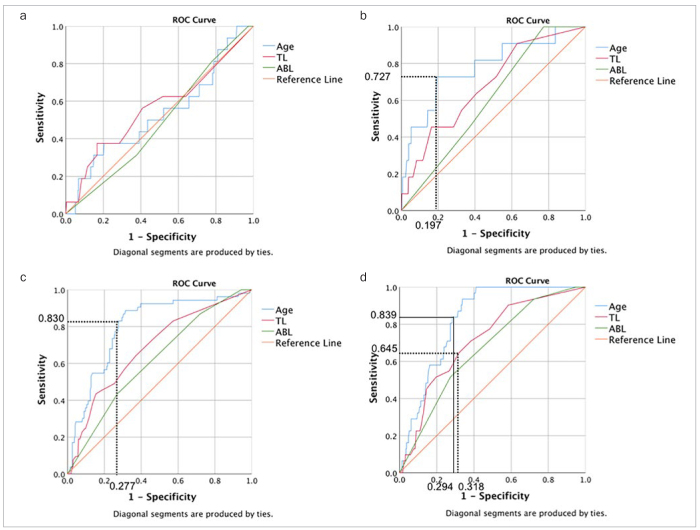
ROC curves and AUCs: (A) ROC curves and AUCs to identify patients with a 3 mm MCW based on age, TL and ABL in male. There was no statistically significant relationship between the parameters and the diagnostic threshold of a 3 mm MCW. (B) ROC curves and AUCs for identifying patients with a MIC Class 3 based on age, TL and ABL in males. AUC for age was interpreted as moderately accurate. The concrete cut-off value for age to identify patients with a MIC Class 3 was 72.55 years. (C) ROC curves and AUCs for identifying patients with a 3 mm MCW based on age, TL and ABL in females. AUC of age was interpreted as moderately accurate. The concrete cut-off value for age to identify patients with a 3 mm MCW was 60.15 years. (D) ROC curves and AUCs for identifying patients with a MIC Class 3 based on age, TL and ABL in females. AUCs for age and TL were interpreted as moderately accurate. The concrete cut-off values for age and TL to identify patients with a MIC Class 3 were 61.55 years and 3.5 lost teeth, respectively.

In males, there was no statistically significant relationship between the parameters of age, TL, or ABL and a diagnostic threshold of 3-mm MCW (Table 5, Fig 4a). Among males, the AUC to identify patients with MIC class 3 based on age, TL and ABL, was statistically significant only for age. The concrete cut-off values for age for identifying patients with MIC class 3 was 72.55 years (Fig 4b).

**Table 5:** AUC of the parameters as probable predictors at a 3-mm MCW and MIC 3

		Diagnostic threshold for the risks for osteoporosis					
		3 mm MCW			MIC 3		
		Area	Asymptotic 95% confidence interval	Asymptotic significance	Area	Asymptotic 95% confidence interval	Asymptotic significance
Age	Male	0.529	0.373 - 0.685	p = 0.694	0.779	0.626 - 0.932	p = 0.002
	Female	0.799	0.735 - 0.863	p = 0.000	0.826	0.774 - 0.878	p = 0.000
TL	Male	0.563	0.401 - 0.725	p = 0.399	0.683	0.529 - 0.837	p = 0.039
	Female	0.683	0.605 - 0.762	p = 0.000	0.715	0.625 - 0.803	p = 0.000
ABL	Male	0.488	0.352 - 0.623	p = 0.868	0.604	0.467 - 0.741	p = 0.467
	Female	0.617	0.538 - 0.696	p = 0.007	0.667	0.575 - 0.758	p = 0.002

In females, the AUC for age was the only parameter to identify patients with 3-mm MCW with at least a moderate accuracy (Table 5). The concrete cut-off value for age to identify patients with 3-mm MCW value was 60.15 years (Fig 4c). For MIC class 3, AUCs of age and TL, interpreted moderately accurately, were 0.826 and 0.715, respectively. The concrete cut-off values for age and TL to identify patients with MIC class 3 were 61.55 years and 3.5, respectively (Fig 4d).

## Discussion

### Mandibular Radiomophormetric Indices and Their Relation to Age and Gender

MCW and MIC values from a large sample of male and female dental patients of various ages were measured in the present study using panoramic images and commercially available software (artificial intelligence). In the current study, MCW and MIC values obtained from panoramic images were not corrected for the magnification factor. Therefore, our MCW values may be slightly overestimated, due mainly to the panoramic machine. The data obtained allowed an assessment of how, if at all, the subject’s age, gender, tooth loss or alveolar bone loss were correled with MCW and MIC, in terms of a potential relation to osteoporosis diagnosis. The usefulness and reliability of MCW and MIC for osteoporosis screening has previously been validated by correlations between MCW and/or MIC and BMDs in the hip and spine, bone turnover rate, and risk of bone fractures.^4,23,30,33,34^ Furthermore, the evaluation of the mandibular inferior cortex by using MCW and MIC can be useful not only for detecting osteoporotic elderly men and women, but also for identifying paramenopausal women and younger individuals at risk of osteoporosis.^31^

In the present study, characteristic changes were observed in the size of MCW and frequency of MIC classes according to gender and age groups. For MCW, a statistically significant correlation with age was seen in females, but not in males. The size in females was larger than that in males in the age groups of 20s, 30s, and 40s. MCW in females increased with age until the 40s, then sharply decreased in the age group of 50s, at which menopause usually occurs. In contrast, MCW in males increased gradually until their 60s, and gradually decreased in size thereafter. A study in a Japanese population showed that the average MCW value for females gradually decreased after the age of 50, and that in their 60s, it was statistically significantly lower than that in their 50s.^32^ A population from Laos showed decreasing MCW values in the age group of 50-59 years for females.^27^ A study from the United States reported that the Mental Index (MI) – a comparable parameter to the MCW value used here – decreased with age group in female subjects from the age of 40 to 79.^7^ The thinning of the MCW during the 50s is considered to be an effect of menopause, and this results in a statistically significant gender difference in the age groups of 60s, 70s, and 80s, in which the MCW in females was significantly lower than in males.

A statistically significant gender difference was observed for the MIC classes, specifically in the age group of 80s for class 1, and 60s, 70s, and 80s for class 3. The frequency of class 1 decreased gradually in males according to the age group. In contrast, a statistically significant decrease was found in females in the age groups of 60s and 80s, generally postmenopausal ages. The frequency of class 3 increased in the older age groups. A study in a Turkish population found that class 1 was less common in people in their 70s and older, and class 3 increased with age in both genders.^5^ They also found females as more frequently having a class 3 than males. Another study from Turkey reported that class 2 was the most common in all age groups in males, and more common than in females.^9^ The results from a British study involving younger females also demonstrated that type C2, which is comparable to class 2 in the present study, was frequently detected in the age range from 25 to 39 years.^18^ It may seem unusual to detect an eroded mandibular inferior cortex in people in their 20s, because it is said that the bone mass has a peak in the late teens and early 20s.^36^ The MIC values in the present study were automatically evaluated and provided by the software used. According to Horiba et al,^10^ the classification of class 2 is challenging because of the absence of a clear distinction from classes 1 and 3.^10^ Ariji et al^2^ clarified the main cause for disagreement of diagnosis between classes 1 and 2 was the slight resorption at the endosteal margin with sufficient thickness of cortex.

### Correlation Between Mandibular Radiomorphometric Indices and Parameters for Periodontal Condition

TL (total number of missing teeth) and ABL (the degree of alveolar bone loss) were used as periodontal parameters to assess the potential relation with MCW and MIC values in the present study. While neither TL nor ABL in males showed any correlation with the MCW, TL in females exhibited a weak correlation. The MCW rapidly decreased after the 50s age group and TL increased sharply from the age of 60s to 80s. Taguchi et al^32^ concluded that a relation between osteoporosis or osteopenia and tooth loss was evident for postmenopausal women. This seems to be consistent with the present results.

Age, TL and ABL were listed in decreasing order of the correlation coefficients for a potential correlation with MIC grading. For TL, more missing teeth were observed in subjects with a more highly eroded mandibular cortex in both genders of the present study. Gulsahi et al^9^ reported that the likelihood of being in Class 3 for edentulous subjects was 27.30 times higher than those for dentate. These results seem to be consistent with the ones in the present study. Concerning ABL, Juluri et al^12^ found no correlation between BMD and ABL. Although Tazel et al^35^ reported a statistically significant correlation between these two factors in 70 postmenopausal women, the correlation may not be very strong. The current study showed a statistically significant but weak correlation between ABL and MIC grades for both genders. Thus, it may be concluded that marginal alveolar bone loss may not be affected by osteoporotic signs in the mandible or systemic osteoporosis.

The usefulness of quantitative and qualitative indices for MCW and MIC for osteoporosis screening has been demonstrated in previous studies,^4,28,29,30,34^ and a diagnostic threshold for screening the risk of osteoporosis has been proposed.^4,6,28,34^ Devlin et al^6^ concluded that patients with a MCW < 3 mm have a high risk of osteoporosis. Meanwhile, Bollen et al^4^ and Taguchi et al^28,34^ found that women with class 3 MIC had a high risk of osteoporosis. Therefore, the present study used 3 mm for the MCW and class 3 MIC as the diagnostic threshold for osteoporosis to look for influencing parameters among age, TL and ABL to help identify patients at risk of osteoporosis.

Two different cut-off values for age to identify patients with a risk of osteoporosis were obtained in females according to the diagnostic threshold mentioned above: 60.15 years at the first threshold (3-mm MCW), but 61.55 years at the second (class 3 MIC). This difference of 1.5 years between the two radiomorphometric indices seems minimal. One potential reason for this difference might be the screening accuracy, because sensitivity for MCW is higher than for MIC.^30^ A study evaluated the prevalence of osteoporosis in postmenopausal Chinese women in Hong Kong.^19^ The study revealed the mean age of women with osteoporosis was 59.7 years based on the T-score of the spine. Their results showed almost the same age as the one for the 3-mm MCW cut-off value used in the present study.

The present study suggested that patients with more than 3.5 missing teeth may be at risk of osteoporosis. A study in south Indian postmenopausal women described that the average number of teeth lost was 5.4 ± 2.8, and it was statistically significantly higher in the osteoporosis group.^14^ If this number also counted wisdom teeth, the ratio of tooth loss was somewhat higher than our 3.5 lost per 28 teeth. A Korean study which evaluated an association between BMD and teeth present analysed the difference in the number of existing teeth between two age groups in osteoporosis.^16^ The age group of 50-64 years with osteoporosis had about 24 teeth, which seems similar to the data in the present study. Also, in the current investigation, TL in females sharply increased from 2.7 to 6.1 teeth on average between age groups 50s and 60s.

## Conclusion

Based on the present findings, relevant indicators for a risk of osteoporosis in Chinese females are age group early 60s and more than 3.5 missing teeth. These clinical signs may justify a further assessment to selectively identify potentially osteoporotic patients, and refer them for further specialist evaluation. It might be recommendable to assess panoramic views of females over the age of 60 and males over the age of 70 for MIC Class 3 by means of an artificial intelligence-based software as used here.
